# Identifying the influencing factors for cataract surgery uptake in Malaysia

**DOI:** 10.1371/journal.pone.0319123

**Published:** 2025-09-12

**Authors:** Mohamad Aziz Salowi, Nyi Nyi Naing, Ju Fan Tay, Wan Radziah Wan Nawang, Siti Nurhuda Sharudin, Norasyikin Mustafa, Nor Fariza Ngah

**Affiliations:** 1 Clinical Research Centre, National Institute of Health, Shah Alam, Selangor, Ministry of Health, Malaysia; 2 Faculty of Medicine, Universiti Sultan Zainal Abidin, Kuala Terengganu, Terengganu, Malaysia; 3 Clinical Research Centre, Selayang Hospital, Batu Caves, Selangor, Ministry of Health, Malaysia; 4 Department of Ophthalmology, Selayang Hospital, Batu Caves, Selangor, Ministry of Health, Malaysia; 5 Faculty of Medicine, Universiti Teknologi MARA, Sungai Buloh, Selangor, Malaysia; 6 Deputy Director General Office (Research and Technical Support), Ministry of Health, Putrajaya, Malaysia; UCMI: University College MAIWP International, MALAYSIA

## Abstract

**Background/Aims:**

In 2014, Malaysia conducted National Eye Survey II (NES II) using the World Health Organization (WHO)-recommended Rapid Assessment of Avoidable Blindness (RAAB) methodology across six administrative regions. The survey revealed significant discrepancies in key national eye care indicators, including prevalence of blindness, cataract surgery outcome, and effective Cataract Surgical Coverage (eCSC). In response, the Ministry of Health (MOH) launched the *Klinik Katarak Kementerian Kesihatan Malaysia* (*KK-KKM*) initiative, which includes mobile cataract surgical services to improve equitable access in the underserved areas in Sarawak and the Eastern Region. Despite endorsement and consistent operational funds, variations in cataract surgery uptake between regions persisted. We postulate that this disparity could be due to community-related factors and their interactions. In 2023, follow-up surveys were conducted in both regions to compare outcomes over time. The eligible subjects were consecutively recruited for a questionnaire interview. This study aims to identify the factors influencing cataract surgery uptake in Malaysia, specifically in Sarawak and Eastern Region.

**Methods:**

A cross-sectional survey was carried out in 2023 using RAAB methodology, targeting individuals aged 50 years and above. Subjects with operable cataract with Pinhole Visual Acuity worse than 6/18, or a history of cataract surgery, were interviewed using the validated 22-item CatSurg-U questionnaire focused on Knowledge, Perception, Attitude and Practice. A total of 1,119 respondents (Sarawak: 408; Eastern 711) were recruited from 203 clusters. Binary logistic regression was used to identify demographic, socioeconomic and other predictors associated with non-uptake of cataract surgery.

**Results:**

The identified factors that influenced individuals with unilateral operable cataracts to have “no surgery” in Sarawak included their “perception to own sight” [AOR: 0.67, 95% CI (0.53, 0.84) ***P*** = 0.001] and “attitude towards treatment” [AOR:1.47, 95% CI (1.17, 1.85) ***P*** = 0.001]. Meanwhile, in the Eastern region, the factors were “perception to own sight” [AOR: 0.80, 95% CI (0.69, 0.92) ***P*** = 0.002], “attitude towards treatment” [AOR:1.15, 95% CI (1.03, 1.29) ***P*** = 0.016], and “practice towards information” [AOR: 1.23, 95% CI (1.01, 1.50) ***P*** = 0.042]. For subjects with bilateral operable cataract in Sarawak, the factors that influenced them to have “no surgery” were “knowledge on surgery” [AOR: 0.35, 95% CI (0.25, 0.50) ***P*** < 0.001], “perception to own sight” [AOR: 1.48, 95% CI (1.15, 1.89) ***P* ** = 0.002], ethnicity (Chinese compared to Malays) [AOR: 0.19, 95% CI (0.04, 0.88) ***P*** = 0.033] and education level (primary school compared to secondary school or above) [AOR: 5.54, 95% CI (1.49, 20.69) ***P* ** = 0.011]. Additionally, for the Eastern region, the factors identified were “knowledge on surgery” [AOR: 0.35, 95% CI (0.26, 0.48) ***P*** < 0.001] and “practice on surgery” [AOR: 0.72, 95% CI (0.62, 0.84) ***P*** < 0.001].

**Conclusion:**

“Perception to own sight”, “attitude towards treatment”, and “knowledge on surgery” were key factors in both regions. Additional barriers in Sarawak include ethnicity and education levels, while in the Eastern Region, “practice towards information” and “practice on surgery” were key factors. Addressing these factors through targeted strategies such as enhancement of mobile outreach programs, public education, and service capacity expansion is essential. Collaborative efforts are needed to improve eye care delivery and ensure equitable access nationwide.

## Introduction

Malaysia signed the World Health Assembly resolution (WHA66.4: Towards Universal Eye Health: A Global Action Plan 2014–2019) during the 66^th^ WHA Geneva in May 2013. By signing the documents, the country pledged its commitment to implementing the items in the resolution and monitoring its progress [1,2]. In alignment with this commitment, a national-level initiative was undertaken to establish baseline data on key eye health indicators, aimed at guiding planning efforts, improving service delivery, and ensuring equitable access to eye care. Consequently, the National Eye Survey II (NES II) was conducted in 2014. Utilising the World Health Organization (WHO)-recommended Rapid Assessment of Avoidable Blindness (RAAB) methodology, the survey was implemented simultaneously across six administrative regions of Malaysia: Central, Eastern, Northern, Southern, Sabah and Sarawak.

NES II results revealed significant regional disparities in the prevalence of blindness. The Central region reported the lowest rate at 0.5%, followed by the Southern region at 0.9%. Higher prevalence was recorded in the Eastern (1.4%) and Northern (1.5%) regions, with the highest in Sabah (1.9%) and Sarawak (1.6%). In terms of cataract surgical outcomes, only 73.0% of patients in Sarawak and 73.2% in Sabah achieved good vision (6/12 or better) after surgery. The Eastern region had 70.6%, Southern 76.7%, Northern 79.1%, and the Central region recorded the best outcome at 81.9%. At the 3/60 threshold for effective Cataract Surgical Coverage (eCSC), Sabah recorded the lowest rate at 51.8%, followed by the Eastern region (57.4%), Sarawak (61.8%), Southern region (66.7%), Northern region (70.5%), and the Central region with the highest coverage at 81.1% [3].

**Fig 1:**
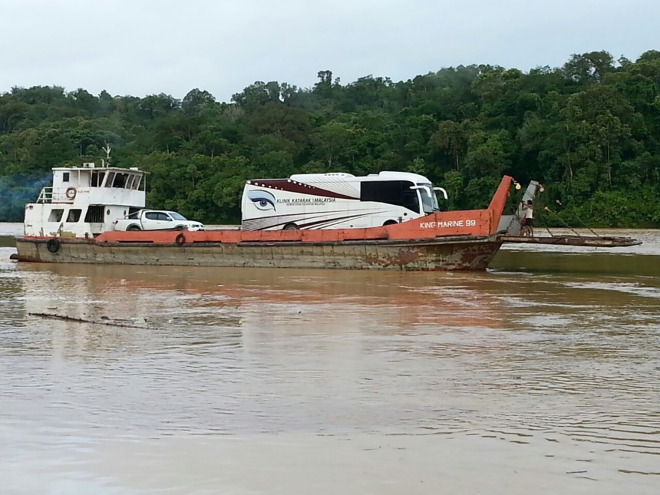
The *KK-KKM* mobile unit en route by barge to the remote district of Kapit in Sarawak.

Based on the discrepancies of key findings between the administrative regions during the NES II, the government launched *Klinik Katarak-Kementerian Kesihatan Malaysia* (Cataract Clinic Ministry of Health Malaysia, *KK-KKM*) as a community cataract pilot project in Sarawak (the Malaysian Borneo) and the Eastern Region of Peninsular Malaysia aiming to address the issue of high prevalence of blindness and cataract blindness [Fig 1]. The objective, concept and operational strategies of the program were subsequently audited and endorsed through the Case Study process of the WHO Western Pacific Innovation Challenge initiative in 2021/2022 [4].

In 2023, nearly a decade after the implementation of NES II, a follow-up survey was conducted in Sarawak and Eastern Region – areas where the *KK-KKM* mobile outreach service had been continuously deployed. The results demonstrated substantial improvements, with a notable decline in the prevalence of blindness: Sarawak reported a reduction to 0.6% and the Eastern region to 0.8% [5]. Visual acuity outcomes following cataract surgery also improved markedly, with 89.8% in Sarawak and 87.5% in the Eastern region achieving good visual outcomes (6/12 or better) [6]. Furthermore, effective cataract surgical coverage (eCSC) at the 3/60 threshold had increased to 83.8% in Sarawak and 74.4% in the Eastern region [7]. These positive trends indicate progress but also underscore the need for continued, targeted efforts to reduce regional disparities and ensure equitable access to quality eye care services nationwide.

Despite the establishment of service brands and the allocation of specific funds to the *KK-KKM* project, there remains a disparity in hospital and community-based data pertaining to cataract surgery (including patient profiles, surgeon practices, and key indicators for service performance and uptake) between the two regions, with the Eastern Region constantly underperforming [8–10].

We aim to analyse the association between the predictors and factors with the outcome (surgery done and surgery not done) in the community during the follow-up population survey after nine years of *KK-KKM* implementation in Sarawak and the Eastern Region. The findings from the study could potentially be used to formulate new policies or strengthen and optimise the implementation of the *KK-KKM* program, hence addressing the issue of service outcome discrepancy between the regions.

## Materials and methods

This was a cross-sectional population survey using the Rapid Assessment of Avoidable Blindness (RAAB) technique to obtain data on eye health, specifically focusing on cataract surgery status. Additionally, structured interviews were conducted through a questionnaire to identify various factors that may influence surgical status, including knowledge, perceptions, attitudes, practices, as well as demographic and socioeconomic characteristics.

This study was conducted in two phases. Phase 1 involved the development and validation of a questionnaire. The factors and questionnaires were developed from an extensive literature search, expert brainstorming sessions, and evaluation of content and face validity. Content Validity Index (CVI) and Face Validity Index (FVI) were calculated at the initial phase of the questionnaire development. For the original 51-item questionnaire, low scale-level CVI values ranged from 0.50 to 0.71, and low item-level CVI values ranged from 0.17 to 0.67. These corresponded to eight items that were subsequently removed. After item removal, the mean item-level CVI was 0.98, and the average scale-level CVI was 0.93. Similarly, scale-level FVI values ranged from 0.55 to 0.78; and item-level FVI values from 0.30 to 0.70, leading to removal of seven items.

This process was followed by validation and reliability analysis using exploratory factor analysis (EFA), reliability tests, confirmatory factor analysis (CFA) and test-retest to produce a questionnaire named CatSurg-U Questionnaire (Cat = Cataract; Surg = Surgery; U = Uptake). It contained six factors and 22 questionnaire items [Table 1]. Cronbach’s alpha values of individual factors ranged from 0.50 to 0.72, and the total Cronbach’s alpha for the final model (22 items) was 0.72. CFA revealed a moderate absolute and reasonable parsimonious fit model. Additional details on the validity and reliability testing of the CatSurg-U questionnaire are included in a separate manuscript currently under submission.

**Table 1:** Factors and questionnaire items (22 items) – for CatSurg-U data collection.

No	Factor	CatSurg-U Numbering	Question/Response Item	Response Choices
1	Knowledge on Cataract	C1	Cataract is the main cause of visual problem among people above 50 years	1 = Yes 2 = Unsure 3 = No
2		C2	Treatment for blurring of vision can be obtained through any government clinic	
3		C3	Financial aid is available if I cannot afford to pay for the cataract surgery	
4	Knowledge on Surgery	C4	Cataract surgery can be done as daycare	1 = Yes 2 = Unsure 3 = No
5		C5	Cataract surgery can be performed under local anaesthesia	
6		C6	Artificial lens will be implanted during surgery for Cataract	
7*	Perception to own Sight	C7	My current vision is poor	1 = Strongly Agree 2 = Agree 3 = Unsure 4 = Not Agree 5 = Strongly Not Agree
8		C8	My current vision does not affect my daily activities	
9		C9	I accept my present vision as part of natural aging process which does not require treatment	
10		C10	I believe treatment is NOT required for Cataract as long as there is no eye pain	
11*	Attitude towards Treatment	C11	My family/neighbours/friends are available to assist me for cataract surgery	1 = Strongly Agree 2 = Agree 3 = Unsure 4 = Not Agree 5 = Strongly Not Agree
12		C12	Other issues are more important than my sight treatment	
13		C13	I believe more on other treatment for Cataract than surgery	
14		C14	I believe males should be given priority to undergo cataract surgery	
15*		C15	I prefer to have my cataract surgery done at government hospital	
16*		C16	I seek treatment at government clinic	
17*	Practice towards Information	C17	I obtain information about Cataract from television or radio	1 = Always 2 = Often 3 = Seldom 4 = Never
18*		C18	I obtain most information about Cataract from printed reading materials	
19*		C19	I obtain most information about Cataract from internet search and social media (Google/Twitter/Facebook/Instagram/WhatsApp/Telegram/Others)	
20*	Practice on Surgery	C20	I am willing to pay for my cataract surgery at Government hospital	1 = Always 2 = Often 3 = Seldom 4 = Never
21*		C21	I will make my own decision to undergo cataract surgery	
22*		C22	I can prioritize going for surgery on the given date	

C refers to the allocated module in the CatSurg-U Questionnaire

Questions only in English (Questions in *Bahasa Malaysia* were not displayed)

Questions marked * were reverse-coded

Phase 1 was followed by Phase 2, a survey using the RAAB method to obtain population data on eye health and blindness [11,12]. The survey’s methodology, prevalence and cataract surgical outcome have been published [5,6]. Eligible subjects from the same sample were further interviewed using the validated CatSurg-U Questionnaire to identify factors influencing the cataract surgery uptake in both regions.

### Ethics approvals

Ethical approvals were obtained from Medical Research and Ethics Committees (MREC) of the Ministry of Health, Malaysia (Research ID NMRR-19-197-46172). The study was conducted in accordance with the tenets of the Declaration of Helsinki.

### RAAB methodology concept/summary

The Rapid Assessment of Avoidable Blindness (RAAB) is a cost-effective, rapid cross-sectional population survey methodology designed to assess the prevalence and causes of blindness, cataract surgical coverage and outcomes after cataract surgery, specifically targeting individuals aged 50 years and above – the age group most affected by blindness [11]. RAAB deploys a simplified yet effective eye examination protocol and employs multistage cluster sampling, beginning with probability proportionate to size (PPS) at the Department of Statistics Malaysia (DOSM) level. Subsequent sampling of subjects within the clusters, enumeration, examination, and interviews are conducted concurrently in the field and data entry into the RAAB 7 software allows for automated analysis [13]. This method is efficient, requires minimal time and equipment, and can be reliably implemented by trained ophthalmology trainees, paramedics and optometrists. The data collected from the survey serve a crucial role in designing and monitoring eye care programs within the surveyed regions [11,12].

### The sampling frame for the RAAB Survey

Department of Statistics, Malaysia (DOSM), once every ten years, conducts data collection for the National Population and Housing Census. An Enumeration Block (EB), a population unit with 80–100 houses/residents each, is outlined according to the population distribution, based on the latest findings during the survey. This is followed by the corresponding geographical map drawing, indicating the exact location and borders of each EB. The EBs are gazetted for fieldwork activities such as morbidity, nutrition, household expenditure, and labour force surveys [14–16].

For this study, all EBs from the 2020 national census were used to select clusters for the RAAB. A total of 98 EBs for Sarawak and 105 EBs were randomly selected for the Eastern Region regardless of strata, using the Probability Proportionate to Size (PPS) technique. Individual EB codes and the corresponding maps were then brought to the field to be used to locate the EBs for data collection.

### Training for RAAB survey and CatSurg-U questionnaire

Each region had six teams of data collectors comprising three persons: two doctors and one allied health staff member trained in ophthalmology. The training for the survey teams was a comprehensive process, conducted separately in each region one week before the fieldwork. Both sessions were led by the first author, a certified Western Pacific RAAB trainer, to monitor data quality and adherence to the study protocol. The survey team members were required to attend four training days, including RAAB and CatSurg-U Questionnaire lectures, inter-observer or inter-interviewer variation, IOV (inter-interviewer reliability) assessment for both RAAB (IOV RAAB) and CatSurg-U (IOV CatSurg-U) and a pilot survey in one of the nearby EBs during fieldwork. Each region had one coordinator responsible for the survey’s smooth implementation and progress monitoring. IOV assessments were done separately for the RAAB and CatSurg-U Questionnaire. The method and kappa results for IOV RAAB have been addressed in other publications [5–7].

### IOV CatSurg-U

During training, the assessment for IOV CatSurg-U was conducted after the IOV RAAB on subjects who were a mix of hospital staff and patients from the outpatient department. The subjects were different from the recruited subjects of the IOV RAAB. As per the questionnaire, 22 questions were asked by six interviewers representing each survey team. Twenty-two (22) subjects in Sarawak and 20 in the Eastern Region volunteered to be interviewed. The subjects included individuals with mixed normal and impaired vision, including cataracts and pseudophakia (had been operated on for cataracts). They were interviewed by rotation (each subject was interviewed six times using the same questionnaire, starting at different chronological numbers). The agreements were analysed using Fleiss Kappa [17]. For Sarawak, two items had a fair agreement, ten had a moderate agreement, eight had a substantial agreement, and two had an almost perfect agreement. For the Eastern Region, six items had a fair agreement, seven had a moderate agreement, seven had a substantial agreement, and two had an almost perfect agreement. For both regions, C8 and C9 had only fair agreement. All agreements were statistically significant [Table 2]. Similar to the intervention taken after IOV RAAB, post-mortem interviews and group retraining focusing on the disagreements were done before the team went to the field the next day for an actual survey. Each team member was re-explained regarding each question’s aim and trained on the best possible way to administer it to the subjects. The emphasis was also given only to allow the assigned person to conduct the interview throughout the survey.

**Table 2:** Inter-interviewer Reliability Assessment of Six Interviewers of Sarawak and the. Eastern Region for agreement on CatSurg-U Questionnaire; Fleiss Kappa by Response Item (22 Questions).

No	Item from CatSurg-U Questionnaire	Fleiss Kappa	Asymptotic Standard Error	lower 95% CI	upper 95% CI	*P* *-value*
	Sarawak					
1	C1	0.67	0.07	0.53	0.81	<0.001
2	C2	0.60	0.07	0.46	0.74	<0.001
3	C3	0.74	0.06	0.61	0.87	<0.001
4	C4	0.82	0.05	0.71	0.92	<0.001
5	C5	0.73	0.07	0.59	0.86	<0.001
6	C6	0.81	0.06	0.70	0.92	<0.001
7	C7	0.52	0.06	0.41	0.62	<0.001
8	C8	0.38	0.06	0.26	0.50	<0.001
9	C9	0.37	0.06	0.26	0.49	<0.001
10	C10	0.52	0.06	0.40	0.63	<0.001
11	C11	0.58	0.05	0.47	0.69	<0.001
12	C12	0.43	0.06	0.30	0.55	<0.001
13	C13	0.52	0.06	0.40	0.64	<0.001
14	C14	0.63	0.06	0.51	0.75	<0.001
15	C15	0.59	0.06	0.48	0.71	<0.001
16	C16	0.41	0.07	0.28	0.54	<0.001
17	C17	0.68	0.05	0.58	0.79	<0.001
18	C18	0.68	0.05	0.57	0.78	<0.001
19	C19	0.79	0.04	0.70	0.87	<0.001
20	C20	0.62	0.05	0.51	0.73	<0.001
21	C21	0.57	0.06	0.45	0.69	<0.001
22	C22	0.50	0.07	0.36	0.64	<0.001
	Eastern					
1	C1	0.68	0.08	0.52	0.85	<0.001
2	C2	0.67	0.12	0.43	0.91	<0.001
3	C3	0.73	0.08	0.57	0.89	<0.001
4	C4	0.58	0.11	0.37	0.79	<0.001
5	C5	0.84	0.08	0.68	0.99	<0.001
6	C6	0.83	0.08	0.66	0.99	<0.001
7	C7	0.56	0.06	0.44	0.68	<0.001
8	C8	0.25	0.07	0.12	0.39	<0.001
9	C9	0.21	0.07	0.08	0.35	0.002
10	C10	0.37	0.09	0.19	0.55	<0.001
11	C11	0.42	0.09	0.24	0.60	<0.001
12	C12	0.40	0.08	0.24	0.56	<0.001
13	C13	0.26	0.11	0.05	0.48	0.017
14	C14	0.48	0.07	0.34	0.63	<0.001
15	C15	0.57	0.09	0.39	0.75	<0.001
16	C16	0.65	0.08	0.50	0.80	<0.001
17	C17	0.64	0.06	0.53	0.75	<0.001
18	C18	0.64	0.05	0.54	0.75	<0.001
19	C19	0.74	0.05	0.64	0.84	<0.001
20	C20	0.46	0.09	0.28	0.64	<0.001
21	C21	0.53	0.08	0.37	0.70	<0.001
22	C22	0.34	0.11	0.13	0.55	0.001

Fleiss Kappa Interpretation:

0.81 - 1.00: Almost perfect agreement

0.61 - 0.80: Substantial agreement

0.41 - 0.60: Moderate agreement

0.21 - 0.40: Fair agreement

0.00 - 0.20: Slight agreement

Below 0.00: Poor agreement

### Survey methods for RAAB survey

Two cross-sectional, population-based surveys, which adhering to the RAAB protocol, were conducted simultaneously from 1^st^ July to 31^st^ October 2023, to assess the prevalence of blindness and visual impairment and its causes, the prevalence of cataract blindness, visual outcomes after cataract surgery, Cataract Surgical Coverage (CSC), effective Cataract Surgical Coverage (eCSC) and effective Refractive Error Coverage (eREC). Each team surveyed 16–17 randomly selected clusters and examined 50 persons aged 50 years and older in each selected enumeration block (EB). Written informed consent was obtained from all respondents, with verbal consent documented, with a witness present, for subjects who were illiterate. Data collection included demographic, medical, and ocular history, followed by visual acuity assessment using the RAAB 7 application and eye examination with a hand-held ophthalmoscope [12]. Subjects with visual impairment were assessed for their primary cause and referred to the nearest ophthalmic care facility for further management. The details of the RAAB survey protocol and methodology have been described elsewhere [11,12].

### Survey methods for CatSurg-U questionnaire

The CatSurg-U Questionnaire was administered to individuals with visual impairment with Pinhole VA worse than 6/18 due to operable cataract as the main cause, and to those who had undergone cataract surgery, either pseudophakia or aphakia, in one or both eyes, regardless of their visual acuity level. Eligible respondents were consecutively selected based on these criteria, provided they could speak and understand Malay and/or English, had resided in Malaysia for at least six months, and gave informed consent. In addition to standard RAAB variables, supplementary demographic data such as ethnicity, marital status, occupation, education level and monthly income were collected. All responses were anonymised and submitted via Google Forms for centralised compilation [18].

### Sample size calculation

The sample size for the RAAB survey was determined using the dedicated sample size calculator available on the RAAB website. One of the key input parameters was the population aged 50 years and above in each region, based on data from the Malaysian National Census 2020 [14,15]. A blindness prevalence of 1.5% for the Eastern Region and 1.6% for Sarawak, as reported in NES II (2014), was applied in the calculation, along with a 95% confidence interval, cluster size of 50, 30% precision, an estimated design effect (DEFF) of 1.5 and 20.0% possibility of non-responders [11,12]. The calculation yielded a required sample size of 98 clusters (4900 subjects) for Sarawak and 105 clusters (5239 subjects) for the Eastern Region.

The required sample size for the CatSurg-U questionnaire was calculated based on the Event Per Variable (EPV) method, as binary logistic regression was planned to analyse the association of socioeconomic and demographic variables, factors and outcomes. EPV is defined as the ratio of the number of outcome events (no surgery) to the number of predictor variables or factors in the logistic regression model. Following the rule of thumb, 10 events per variable were required to achieve stable and reliable estimates. For example, if there were five (5) predictor variables or factors in the model, and we aimed for a minimum of 10 events per variable, the required number of events (no surgery) would be: required events = 10 × number of predictor variables or factors, therefore required events = 10 × 5 = 50 events [19].

The calculation included participants with operable cataracts, both those who had undergone surgery and those who had not (defined as events). For the logistic regression analysis, the required sample size was determined based on the number of events—specifically, individuals with operable cataracts who had not received surgery.

For this study, the required number of events (no surgery) was calculated separately for unilateral and bilateral eyes and by the region (Sarawak and Eastern Region), to ensure adequate power for subgroup analysis.

The targeted event-based sample sizes were:

Unilateral: Sarawak= 80, Eastern: = 60)

Bilateral: Sarawak= 120, Eastern = 90)

The actual event numbers achieved were:

Unilateral: Sarawak = 37, Eastern = 126

Bilateral: Sarawak = 206, Eastern = 226

As shown, the minimum sample size for unilateral case in Sarawak was not achieved. This is acknowledged as a limitation of the study, as the smaller event count may have reduced the statistical power of the logistic regression model to accurately identify factors influencing cataract surgery uptake within this subgroup.

### Statistical analysis

**Fig 2:**
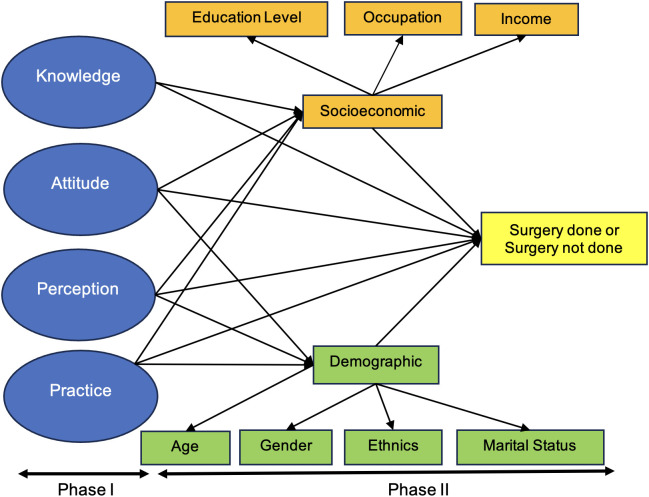
Conceptual framework.

Statistical analysis was performed using IBM SPSS AMOS Statistical Package for Social Science (SPSS), version 26.0 (SPSS, Inc., Chicago, III., USA) for Windows. Inter-interviewer reliability assessment for the CatSurg-U Questionnaire was done using Fleiss Kappa. Binary Logistic Regression was done to identify the association of the predictor variables or factors (demographic, socioeconomic, knowledge, perception, attitude and practice factors) to the dependent outcome (surgery done vs surgery not done with the latter as the outcome event) in one or both eyes (unilateral vs bilateral) [Fig 2]. The analysis started with data exploration and cleaning, followed by simple logistic regression (SLogR) to screen for important independent variables. Variables with ***P**-*value < 0.25 and/or clinically important were selected for multiple logistic regression (MLogR) using forward and backward selection techniques to produce a preliminary main effect model. The modelling procedure was done using the Likelihood Ratio test based on Maximum Likelihood Estimates (MLEs). From SLogR to MLogR, variable selection was done by four major principles: (a) best fit, (b) parsimonious model, (c) biological plausibility, and (d) statistical significance. This step was followed by a multicollinearity & interaction check to produce a preliminary final model. The Hosmer–Lemeshow goodness-of-fit test, classification tables and area under the receiver operating characteristic (ROC) curve were examined to ensure the model’s fitness before the regression model was confirmed. The results were interpreted using crude and adjusted Odds Ratios, 95% confidence intervals and corresponding ***P***-values [20,21].

## Results

Some of the results related to the CatSurg-U Questionnaire development, validation and the RAAB survey are still under review or have been published. This manuscript will only address the CatSurg questionnaire data collection results following the RAAB survey. A total of 1,119 subjects (Sarawak = 408, Eastern = 711) were recruited. The demographic profile of the eligible subjects for the CatSurg-U Questionnaire showed a significantly higher proportion of self-employed females, females with no formal educational background and females with income RM1500 or less per month in Sarawak. There was a significantly higher proportion of Malay females and a higher significant proportion of males with primary school qualifications in the Eastern Region. There was a significantly higher proportion of married males in both regions [Table 3].

**Table 3:** Demographic profile of subjects recruited for the CatSurg-U Questionnaire by gender (Sarawak *n* = 408, Eastern **n* *= 711).

	SARAWAK							EASTERN						
		Female		Male					Female		Male			
	*N*	*n*	%	*n*	%	*χ2Statistic(df)*	*P* *-value*	*N*	*n*	%	*n*	%	χ2Statistic(df)	*P*-value
**Age Group:**														
50-59	32	15	6.5	17	9.7	4.99 (3)	0.173	72	43	10.5	29	9.6	5.41 (3)	0.144
60-69	128	71	30.6	57	32.4			250	143	34.9	107	35.5		
70-79	182	101	43.5	81	46.0			258	138	33.7	120	39.9		
80 and above	32	45	19.4	21	11.9			131	86	21.0	45	15.0		
**Ethnicity (EASTERN):**														
Malay								623	368	90.0	255	85.0	7.97 (2)	0.019
Chinese								65	27	7.0	38	13.0		
Others (including Indian, Bidayuh or Orang Ulu)								23	15	4.0	8	3.0		
**Ethnicity (SARAWAK):**														
Malay	107	58	25.0	49	27.8	5.30 (5)	0.380							
Chinese	73	38	16.4	35	19.9									
Indian	5	2	0.9	3	1.7									
Iban	118	77	33.2	41	23.3									
Bidayuh	34	19	8.2	15	8.5									
Others (including Orang Ulu)	71	38	16.4	33	18.8									
**Marital Status:**														
Married	310	155	66.8	155	88.1	24.78 (1)	<0.001	531	281	68.5	250	83.1	19.35 (1)	<0.001
Unmarried (including divorcee, widow or widower)	98	77	33.2	21	11.9			180	129	31.5	51	16.9		
**Occupation:**														
Government	46	20	8.6	26	14.8	6.78 (2)	0.034	39	19	4.6	20	6.6	5.66 (2)	0.059
Private	18	7	3.0	11	6.3			42	18	4.4	24	8.0		
Self	344	205	88.4	139	79.0			630	373	91.0	257	85.4		
**Level of Education:**														
None	163	115	49.6	48	27.3	35.73 (3)	<0.001	210	156	38.0	54	17.9	40.58 (3)	<0.001
Primary School	146	83	35.8	63	35.8			290	153	37.3	137	45.5		
Secondary School	74	29	12.5	45	25.6			177	91	22.2	86	28.6		
Diploma or above	25	5	2.2	20	11.4			34	10	2.4	24	8.0		
**Average Household Monthly Income:**														
RM1500 or less	196	121	52.2	75	42.6	8.16 (3)	0.043	281	163	39.8	118	39.2	3.51 (3)	0.319
>RM1500-RM3000	129	75	32.3	54	30.7			257	157	38.3	100	33.2		
>RM3000-RM6000	56	24	10.3	32	18.2			135	70	17.1	65	21.6		
>RM6000	27	12	5.2	15	8.5			38	20	4.4	18	5.3		

*df* = Degree of freedom

All were tested using the Chi-Square test.

There were two common significant factors identified which influenced the individuals with unilateral operable cataract to have “no surgery” in both regions (“perception to own sight” and “attitude towards treatment”). The other significant factor was “practice towards information” (specific to the Eastern Region) [Table 4**]**.

**Table 4:** Associated Factors Influencing Individuals with UNILATERAL (one eye) Operable Cataract to have “No Surgery” by Simple and Multiple Logistic Regression Model (Sarawak *n* = 90, Eastern *n* = 212).

	Simple Logistic Regression			Multiple Logistic Regression		
	b	Crude OR (95%CI)	*P*-value	b	Adjusted OR (95%CI)	*P*-value
**SARAWAK:**						
Perception to own Sight	−0.26	0.77 (0.64, 0.94)	0.010	−0.41	0.67 (0.53, 0.84)	0.001
Attitude towards Treatment	0.22	1.24 (1.04, 1.49)	0.019	0.39	1.47 (1.17, 1.85)	0.001
^a^Forward LR Multiple Logistic Regression model was applied Hosmer Lemeshow test (*p* = 0.114), classification table (overall correctly classified percentage = 71.1%) and area under the ROC curve (78.5%) were applied to check the model fit.						
**EASTERN REGION:**						
Perception to own Sight	−0.18	0.84 (0.74, 0.96)	0.009	−0.23	0.80 (0.69, 0.92)	0.002
Attitude towards Treatment	0.16	1.17 (1.06, 1.29)	0.003	0.14	1.15 (1.03, 1.29)	0.016
Practice towards Information	0.14	1.15 (0.97, 1.38)	0.117	0.21	1.23 (1.01, 1.50)	0.042
^a^Backward Likelihood Ratio Multiple Logistic Regression model was applied Hosmer Lemeshow test (*p* = 0.369), classification table (overall correctly classified percentage = 65.1%) and area under the ROC curve (70.8%) were applied to check the model fit For both Sarawak and Eastern: Multicollinearity and interaction terms were checked and not found b = regression coefficient; CI = confidence interval; OR=odds ratio						

In Sarawak:

1) Individuals with better “perception to own sight” had 33.0% lower odds of having no surgery when they had an operable cataract in one eye [AOR: 0.67, 95% CI (0.53, 0.84) ***P* ** = 0.001, adjusted for “attitude towards treatment”].
2) Individuals with better “attitude towards treatment” had 47.0% higher odds of having no surgery when they had an operable cataract in one eye [AOR:1.47, 95% CI (1.17, 1.85) ***P*** = 0.001, adjusted for “perception to own sight”].

In the Eastern Region:

1) Individuals with better “perception to own sight” had 20.0% lower odds of having no surgery when they had an operable cataract in one eye [AOR: 0.80, 95% CI (0.69, 0.92) ***P* ** = 0.002, adjusted for “attitude towards treatment” and “practice towards information”].
2) Individuals with better “attitude towards treatment” had 15.0% higher odds of having no surgery when they had an operable cataract in one eye [AOR: 1.15, 95% CI (1.03, 1.29) ***P **= *0.016, adjusted for “perception to own sight” and “practice towards information”].
3) Individuals with better “practice towards information” had 23.0% higher odds of having no surgery when they had an operable cataract in one eye [AOR: 1.23, 95% CI (1.01, 1.50) ***P* ** = 0.042, adjusted for “perception to own sight” and “attitude towards treatment”].

For bilateral operable cataract, the common significant factor that influenced the individuals to have “no surgery” in both regions was “knowledge on surgery”. The other significant factors were “perception to own sight”, ethnicity and level of education (specific to Sarawak) and “practice on surgery” (specific to the Eastern Region) [Table 5].

**Table 5:** Associated Factors Influencing Individuals with BILATERAL (both eyes) Operable Cataract to have “No Surgery” by Simple and Multiple Logistic Regression Model (Sarawak *n* = 243, Eastern *n* = 352).

	Simple Logistic Regression			Multiple Logistic Regression		
	b	Crude OR (95%CI)	*P*-value	b	Adjusted OR (95%CI)	*P*-value
**SARAWAK:**						
**Ethnicity:**						
Malay	0	1	<0.001	0	1	0.016
Chinese	−1.72	0.18 (0.07, 0.48)	0.001	−1.67	0.19 (0.04, 0.88)	0.033
Iban	1.02	2.76 (0.79. 9.64)	0.111	1.48	4.41 (0.76, 25.73)	0.100
Others (including Bidayuh, Indian or Orang Ulu)	−0.02	0.98 (0.34, 2.08)	0.973	0.41	1.50 (0.37, 6.04)	0.567
**Level of Education:**						
None	2.50	12.13 (4.62, 31.83)	<0.001	0.31	1.36 (0.32, 5.71)	0.677
Primary School	2.11	8.26 (3.35, 20.38)	<0.001	1.71	5.54 (1.49, 20.69)	0.011
Secondary School or above	0	1		0	1	0.028
Knowledge on Surgery	−0.95	0.39 (0.30, 0.50)	<0.001	−1.05	0.35 (0.25, 0.50)	<0.001
Perception to own Sight	−0.32	1.38 (1.16, 1.63)	<0.001	0.39	1.48 (1.15, 1.89)	0.002
Hosmer Lemeshow test (*p* = 0.480), classification table (overall correctly classified percentage = 93.0%) and area under the ROC curve (93.2%) were applied to check the model fit						
**EASTERN REGION:**						
Knowledge on Surgery	−1.27	0.28 (0.21, 0.38)	<0.001	−1.05	0.35 (0.26, 0.48)	<0.001
Practice on Surgery	−0.57	0.56 (0.50, 0.64)	<0.001	−0.33	0.72 (0.62, 0.84)	<0.001
Hosmer Lemeshow test (*p* = 0.126), classification table (overall correctly classified percentage = 84.4%) and area under the ROC curve (90.4%) were applied to check the model fit For both Sarawak and Eastern: ^a^Forward Likelihood Ratio Multiple Logistic Regression model was applied Multicollinearity and interaction terms were checked and not found b = regression coefficient; CI = confidence interval; OR=odds ratio						

In Sarawak:

1) Individuals with better “knowledge on surgery” had 65.0% lower odds of having no surgery when they had operable cataract in both eyes [AOR: 0.35, 95% CI (0.25, 0.50) ***P*** < 0.001, adjusted for ethnicity, level of education and “perception to own sight”].
2) Individuals with better “perception to own sight” had 48.0% higher odds of having no surgery when they had operable cataract in both eyes [AOR: 1.48, 95% CI (1.15, 1.89) ***P*** = 0.002, adjusted for ethnicity, level of education and “knowledge of surgery”].
3) Chinese individuals had 81.0% lower odds, compared to Malay individuals, of having no surgery when they had operable cataract in both eyes [AOR: 0.19, 95% CI (0.04, 0.88) ***P*** = 0.033, adjusted for level of education, “knowledge of surgery” and “perception to own sight”].
4) Individuals who attended primary school had 5.5 times higher odds, compared to those who attended secondary school or above, of having no surgery when they had operable cataract in both eyes [AOR: 5.54, 95% CI (1.49, 20.69) ***P* ** = 0.011, adjusted for ethnicity, “knowledge of surgery” and “perception to own sight”].

In the Eastern Region:

1) Individuals with better “knowledge on surgery” had 65.0% lower odds of having no surgery when they had operable cataract in both eyes [AOR: 0.35, 95% CI (0.26, 0.48) ***P*** < 0.001, adjusted for “practice of surgery”].
2) Individuals with better “practice on surgery” had 28.0% lower odds of having no surgery when they had operable cataract in both eyes [AOR: 0.72, 95% CI (0.62, 0.84) ***P*** < 0.001, adjusted for “knowledge of surgery”].

## Discussion

The KK-KKM project in both regions emphasises scheduled trips for screening and surgery and revisiting them after one month by optometrists to assess patients’ visual outcomes. The timetable for the mobile unit is distributed to all the Provincial Hospitals at the beginning of each calendar year. The fixed schedule allows people in remote areas to plan their finances and trips to come forward and seek eye treatment. Operating in proper operating rooms using standard cataract extraction techniques, adhering to the quality measurement of biometry and fixing the timetable for the service maximises access and ensures quality surgery for the people.

Like all other hospital facilities in the Malaysian Ministry of Health, data from cataract surgeries performed at the *KK-KKM* locations are entered into the National Eye Database, a web-based password-protected surveillance system collecting data on eye diseases and the clinical performance of the ophthalmology program in Malaysia. It consists of online systematic data entry according to predefined sets of preoperative, operative and outcome forms. Details on the Malaysian Cataract Surgery Registry and Cumulative Summation (CUSUM) techniques in cataract surgical performance monitoring have been published elsewhere [22,23]. The main reason behind these strategic measures was to ensure maximum access to quality cataract surgeries for every individual in the region, regardless of differences in socioeconomic and demographic profiles.

This study combined the RAAB survey findings (surgery status obtained from ocular examination by trained eyecare providers) with the CatSurg-U Questionnaire findings (factors and response/questionnaire items), demographic and socioeconomic data [**Fig 2**]. Peng et al. (2013), in their population survey to evaluate the utilisation of eye care services in a rural population in North China, used the frequency of visits to eye care providers as the outcome of multiple logistic regression in analysing the factors associated with the underuse of services [24]. Li et al. (2020), in a cross-sectional survey in rural Yueqing, Wenzhou, China, used a questionnaire on knowledge, attitudes and practices (KAP). Visual acuity (unilateral vs bilateral) was used as the outcome variable of multiple logistic regression to identify factors affecting the use of eye care services [25]. To the best of our knowledge, no available study in the literature yet evaluates the uptake of cataract surgery using an objective outcome measurement (surgery status) obtained from ocular examination.

In 1998, WHO defined a good visual outcome after cataract surgery as achieving visual acuity of ≥6/18, while an outcome of <6/60 was considered poor. It was recommended that at least 80% of patients should attain a good outcome without correction, and 90% with best-corrected visual acuity, with poor outcomes kept below 5%. In 2021, WHO updated these benchmarks, redefining a good surgical outcome as achieving presenting visual acuity of ≥6/12, and setting the eligibility threshold for cataract surgery at best-corrected visual acuity (BCVA) of <6/12 [26].

The following were the outcome variables collected during the fieldwork (RAAB survey) obtained from the ocular examination. They were evaluated and discussed with the research committee before the commencement of the survey:

I Lens status

This variable was obtained from direct eye examination using a handheld direct ophthalmoscope. The findings included normal, opacity, aphakia, pseudophakia with no posterior capsular opacity, and pseudophakia with posterior capsular opacity or no view. Cataract surgery uptake was represented by either aphakia, pseudophakia (surgery done), or opacity (surgery not done).

- Distant Pinhole visual acuity (PinVA)

This variable was obtained by testing the subjects’ distant visual acuity with pinholes using the built-in chart in the RAAB7 apps. The findings were an option of the levels of PinVA (6/12, 6/18, 6/60, 3/60, 1/60, Perception of light or No perception of light). The inclusion criteria for the CatSurg Questionnaire interview was Pinhole VA worse than 6/18.

III Cataract surgery

Four sub-variables represented this variable:

a) Age when the surgery was done (numerical)
b) Type of cataract surgery [categorical: options were no Intraocular Lens (IOL), IOL implantation or couching]
c) Cost of cataract surgery (categorical: options were totally free, partially free or fully paid)
d) Cause of poor vision after cataract surgery (categorical: options were ocular co-morbidity, operative complication, refractive error or long-term complications)

Unlike Variable II and III, Variable I had a clear binary definition between “surgery done” and “surgery not done”. For Variable II, subjects could have undergone surgery, but the visual acuity remained poor (e.g., surgery with intra-operative or post-operative complication); likewise, they could have lens opacity, but the visual acuity remained good. Variable III only captured subjects who had undergone cataract surgery (and excluded subjects who had cataracts but did not undergo surgery).

The results from the multiple regression analysis suggested that there were two common significant factors identified, which influenced the individuals with unilateral operable cataract to have “no surgery” in both regions (“perception to own sight” and “attitude towards treatment”). The other significant factor was “practice towards information” (specific to the Eastern Region). For bilateral operable cataract, the common significant factor that influenced the individuals to have “no surgery” in both regions was “knowledge on surgery”. The other significant factors were “perception to own sight,” ethnicity, and level of education (specific to Sarawak), as well as “practice on surgery” (specific to the Eastern Region).

In Sarawak, having better “perception to own sight” was associated with lower odds of “no surgery” in unilateral cataract. However, the factor was associated with higher odds if the person had a bilateral cataract. The seemingly contradictory role of the “perception to own sight” between unilateral and bilateral operable cataracts can be explained by differences in individuals’ visual awareness and adaptive behaviour. For individuals with unilateral cataract, the disparity in vision between the affected and unaffected eye is often noticeable and functionally limiting, which increases the likelihood of seeking surgical intervention. However, for those with bilateral operable cataracts, vision typically deteriorates gradually in both eyes, leading individuals to adapt to their reduced vision over time. This adaptation, combined with factors such as slow cataract progression, limited daily visual demands, cultural beliefs, and barriers related to access and cost, may result in a lower perceived need for surgery. As a result, the odds of not undergoing surgery are higher in this group despite similar or greater levels of visual impairment.

In Sarawak, where geographical and financial access could be an issue for the community, those with bilateral operable cataract should not be neglected and must be reached out to by expanding the coverage of the mobile unit.

In both regions, when a person had unilateral operable cataract, although a better “perception of own sight” was associated with lower odds of “no surgery”, the opposite was revealed for the “attitude towards treatment”. Even if the community had a positive attitude towards seeking cataract treatment, the odds of having “no surgery” were higher. They had good self-awareness about poor vision in one eye and possibly wanted to seek treatment, but access to treatment (geographical or financial) could be the limiting factor. Interpreting the “attitude towards treatment” and “practice towards information” together, within the scope of the questionnaire items (the better the attitude and practice, the higher the odds of “no surgery) in the Eastern Region pointed more to possible limited “supply” of service despite higher “demand”. A concerted effort to increase the coverage and capacity of services must be addressed to increase the uptake of cataract surgery in the population, especially in the Eastern Region.

Ethnicity (Chinese, compared to Malays) and level of education (primary school, compared to secondary school or above) contributed to the factors influencing individuals in Sarawak with bilateral operable cataract to have “no surgery”. These factors were not identified in the Eastern Region. Sarawak is a multicultural state. According to the 2020 Population and Housing Census, the Iban are the largest indigenous group in Sarawak, comprising approximately 30.0–35.0% of the population, Chinese 24.0–26.0%, Malays 20.0–25.0%, Bidayuh 6.0–7.0% and other indigenous groups, including the Orang Ulu, Melanau, Kayan, Kenyah, Kelabit, and Penan, who together make up around 10.0–15.0% of the population [27]. The Iban are mainly concentrated in rural and inland areas such as Kapit, Sri Aman, and Betong. The Bidayuh predominantly reside in the highland areas of Kuching, Bau, and Serian. Malays and Melanaus are more commonly found along the coastal regions, particularly around Kuching, Mukah, and Miri. The Chinese population is largely based in urban centres like Kuching, Sibu, Bintulu, and Miri. Smaller indigenous groups, including the Orang Ulu, are mostly located in the remote interior of northern Sarawak [27]. The lower odds among Chinese with bilateral operable cataract with “no surgery” perhaps was contributed by the ethnic composition in the state and their better access to treatment (geographically). Analysis of other specific ethnics was not significant. The individuals with primary school qualifications had 5.5 times higher odds of “no surgery “ than those with higher qualifications. These findings emphasised the importance of education in providing eye care in Sarawak and other regions.

A better “knowledge on surgery” if the individuals had bilateral operable cataract was associated with lower odds of “no surgery” in both regions. This indicated that if the community had substantial knowledge about cataract surgery, the odds of them coming forward to seek treatment would be higher. Similarly, “practice on surgery” was associated with lower odds of “no surgery” when the individuals had bilateral operable cataract, but only specific to the Eastern Region.

The strategies proposed to increase the uptake of cataract surgery in the community shall be targeted at the identified influencing factors associated with higher odds of “no surgery” in specific regions. In Sarawak (“attitude towards treatment” in unilateral operable cataract) and (level of education and “perception to own sight” in bilateral operable cataract). In the Eastern Region (“attitude towards treatment” and “practice towards information” in unilateral operable cataract)

Peng et al. (2013), in their evaluation of the use of eye care services in a rural population, revealed ocular comorbidities (glaucoma, age macular degeneration or refractive error) as the factors influencing the community to visit an eye care provider [24]. Li et al. (2020) stated “no need” and “schedule conflicts” as the main reasons for not seeking eye care. High education levels, older age, self-perceived vision condition and regular vision check behaviour were related to seeking eye care services [25].

The remedial measures for this study shall include expanding and upgrading cataract surgical services in both regions. This expansion/upgrade must include the hospital infrastructure, the mobile unit, and workforce capacity, especially the surgeons’ training. This expansion will allow active case detection, especially for individuals residing in remote areas with bilateral operable cataracts. Other remedial measures include strengthening community advocacy, education and engagement to empower and sustain the long-term project, especially in the Eastern Region.

### Limitation

The primary population survey in this study was NES III using the Rapid Assessment of Avoidable Blindness (RAAB) methodology. It collected eye health data (including lens and surgery status). Based on this survey, the sample size was calculated, and grants were allocated. Data collectors extracted the eligible subjects from each randomly selected Enumeration Block (EB) in NES III to be interviewed using the CatSurg-U Questionnaire. The number of subjects, therefore, depended on the availability of eligible subjects within the selected EB. Although calculated, recruiting subjects to satisfy the sample size for the intended regression analysis was difficult, as the population was not sampled for the questionnaire.

## Conclusion

The common factors influencing the cataract surgery uptake in Sarawak and Eastern Region are “perception to own sight”, “attitude towards treatment”, and “knowledge on surgery”. In Sarawak, ethnicity and education levels further influence the uptake. While in Eastern Region, “practice towards information” and “practice on surgery” factors were key barriers. Addressing both common and region-specific factors through targeted strategies, including the enhancement of mobile cataract surgery services, strengthening of public education and expansion of service capacity. Collaborative efforts and further research are needed to improve eye care delivery and ensure equitable access nationwide.

These efforts are vital to improving the uptake of cataract surgery and ensuring equitable access to eye care across the country. In particular, the Eastern Region would benefit from increased investment in infrastructure and service delivery. Collaborative engagement with relevant stakeholders is essential to raise public awareness and knowledge about cataract surgery, while further research among the eye care providers is required to uncover the barriers in the country’s delivery of cataract surgery services.

## Supporting information

S1 Data Raw data. (XLSX)
